# Hospital care direct costs due to ambulatory care sensitive conditions related to diabetes mellitus in the Mexican public healthcare system

**DOI:** 10.1186/s12913-024-10937-w

**Published:** 2024-04-24

**Authors:** Pedro Saturno-Hernández, Estephania Moreno-Zegbe, Ofelia Poblano-Verastegui, Laura del Pilar Torres-Arreola, Arturo C. Bautista-Morales, Cynthya Maya-Hernández, Juan David Uscanga-Castillo, Sergio Flores-Hernández, Patricia María Gómez-Cortez, Waldo Iván Vieyra-Romero

**Affiliations:** 1 National Institute of Public Health (INSP), Cuernavaca, Morelos Mexico; 2 Mexican Consortium of Private Hospitals, Mexico City, Mexico; 3 Mexican Social Security Institute, Mexico City, Mexico

**Keywords:** Health Care costs, Preventable hospitalization, Quality of Health Care, Direct costs

## Abstract

**Background:**

Hospitalizations for ambulatory care sensitive conditions (ACSC) incur substantial costs on the health system that could be partially avoided with adequate outpatient care. Complications of chronic diseases, such as diabetes mellitus (DM), are considered ACSC. Previous studies have shown that hospitalizations due to diabetes have a significant financial burden. In Mexico, DM is a major health concern and a leading cause of death, but there is limited evidence available. This study aimed to estimate the direct costs of hospitalizations by DM-related ACSC in the Mexican public health system.

**Methods:**

We selected three hospitals from each of Mexico’s main public institutions: the Mexican Social Security Institute (IMSS), the Ministry of Health (MoH), and the Institute of Social Security and Services for State Workers (ISSSTE). We employed a bottom-up microcosting approach from the healthcare provider perspective to estimate the total direct costs of hospitalizations for DM-related ACSC. Input data regarding length of stay (LoS), consultations, medications, colloid/crystalloid solutions, procedures, and laboratory/medical imaging studies were obtained from clinical records of a random sample of 532 hospitalizations out of a total of 1,803 DM-related ACSC (ICD-10 codes) discharges during 2016.

**Results:**

The average cost per DM-related ACSC hospitalization varies among institutions, ranging from $1,427 in the MoH to $1,677 in the IMSS and $1,754 in the ISSSTE. The three institutions’ largest expenses are LoS and procedures. Peripheral circulatory and renal complications were the major drivers of hospitalization costs for patients with DM-related ACSC. Direct costs due to hospitalizations for DM-related ACSC in these three institutions represent 1% of the gross domestic product (GDP) dedicated to health and social services and 2% of total hospital care expenses.

**Conclusions:**

The direct costs of hospitalizations for DM-related ACSC vary considerably across institutions. Disparities in such costs for the same ACSC among different institutions suggest potential disparities in care quality across primary and hospital settings (processes and resource utilization), which should be further investigated to ensure optimal supply utilization. Prioritizing preventive measures for peripheral circulatory and renal complications in DM patients could be highly beneficial.

## Background

Hospitalizations for ambulatory care sensitive conditions (ACSC) generate avoidable costs, both economic and human [1–6]. These costs can be driven by several factors, such as resources and accessibility in primary health care (PHC), hospital care, socioeconomic characteristics of the population, and the quality of care itself, which is increasingly recognized as crucial beyond mere service access or utilization [5, 7, 8]. ACSC hospitalizations can be avoided with adequate and timely outpatient care for acute diseases and chronic conditions such as DM [1–6].

ACSC has been used as an indicator of access to and quality of PHC services [9–11] and as a target for cost reduction efforts [12]. Studies have shown variations in ACSC rates [5, 8, 13] and costs [12–15] across and within countries, depending on the ACSC list used and the national health system of each country. Understanding the breakdown of ACSC costs is crucial for identifying drivers and areas where healthcare value can be improved by reducing avoidable expenses without compromising quality [12].

DM, classified as an ACSC [16], is a public health problem that affects 10.5% of the global population (537 million people older than 20 years) [17]. Approximately 643 million people worldwide are expected to have DM by 2030 [18]. In Mexico, the incidence of DM increased from 7 to 9.4% in 10 years (2006–2016) [19, 20]. Mexico ranks among the top ten countries with the highest incidence of diabetes in the adult population and allegedly has the highest total diabetes-related health expenditure [18]. Previous studies indicate that DM is the most common cause of ACSC hospitalization in Mexico [21–23], with a rate nearly double the average of the Organization for Economic Co-operation and Development (OECD) countries (249 and 129 preventable hospitalizations per 100,000 inhabitants, respectively) [10].

With the increasing prevalence of DM, the cost burden of care has also increased [24], particularly for DM-related complications [8]. The limited number of studies that estimated the costs of DM in Mexico has indicated that DM represents a substantial burden on the health system [8, 24–26]. For example, from 2008 to 2013, the Mexican Social Security Institute (IMSS), one of the three main public institutions, incurred a direct cost of $1,563 billion for hospitalizations due to diabetes mellitus. Nearly 83% of this cost was attributed to complications, with an estimated annual cost of $260 million [25]. However, these previous studies utilized diverse methodologies and did not focus on ACSC [24, 26, 27]; instead, they used diagnosis-related groups (DRG) costs [25–27] and cost consensus-building processes but not the actual standard processes of care [24], or included only one of the three main public institutions [8, 24–26]. The estimation of DM-related ACSC direct hospitalization costs based on actual clinical processes and encompassing the three main public institutions is a novel and more comprehensive approach that, to our knowledge, has not been approached so far.

The National Health System in Mexico is segmented into different healthcare subsystems organized under separate national public institutions and private providers/insurers [28, 29]. In the public subsystem, the main institutions are (1) the IMSS, which offers health insurance to formal workers and their families, covering approximately 51% of the population; (2) the Institute of Social Security and Services for State Workers (ISSSTE), providing health insurance for federal government workers, representing 8.8% of the population; and (3) the Ministry of Health (MoH) and the network of public institutions owned and managed by each State, which provide health insurance to poor or uninsured individuals, which covers approximately 35.5% of the population [29, 30].

An information gap exists regarding the direct hospitalization costs for diabetes mellitus-related ambulatory care sensitive conditions (DM/ACSC) and comparisons between Mexico’s three main public healthcare institutions. This study estimated the direct costs of DM/ACSC hospitalizations at the IMSS, ISSSTE, and MoH to compare them and identify potential priority areas for improvement. The findings will help identify potential savings that could be redirected to primary care through targeted strategies to reduce avoidable DM-related hospitalizations.

## Methods

We conducted a multicentric, observational study to estimate the direct costs of hospital care for DM/ACSC from the healthcare provider perspective with a bottom-up microcosting approach [31, 32]. The resources and the amount of each resource used during hospitalization for DM/ACSC were extracted from the clinical records of hospitals in three main public healthcare institutions in Mexico. Our study adheres to the Consolidated Health Economic Evaluation Reporting Standards 2022 framework [33].

### Data source and sample definition

The selection of DM/ACSC hospitalizations was carried out in three steps:

1) Through convenience sampling, three states were selected for this study. One each for the lower, middle, and higher socioeconomic regions of Mexico (Chiapas, Morelos, and Mexico City, respectively), according to the National Institute of Statistics and Geography (INEGI) [34].

2) We selected three hospitals in each State, one per institution (IMSS, ISSSTE, and MoH), based on the higher number of DM/ACSC hospitalizations reported in 2016 by the Department of Health Statistics (DGIS) [35]. DM/ACSC hospitalizations were determined according to the list proposed by Purdy [16] (International Classification of Diseases, ICD-10, E10.0-E10.8 for insulin-dependent DM, E11.0-E11.8 for non-insulin-dependent DM, E12.0-E12.8 for DM associated with malnutrition, E13.0-E13.9 for other specified DM, and E14.0-E14.9 for unspecified DM). 3) The Federal Committees of IMSS and ISSSTE and the State Committees of the Ministry of Health of Chiapas, Morelos, and Mexico City approved the study in their units. They provided the 2016 hospital discharge databases to identify DM/ACSC hospitalizations of adults 18 ≥ years old. To ensure at least 60 DM/ACS hospitalizations with data per hospital, we planned to use a random sample of at least 80 cases per hospital, anticipating up to 25% invalid cases due to misclassification or incomplete data in medical records. This procedure led to the reviewing of all valid clinical records with DM/ACSC hospitalization episodes in some hospitals. Each hospital provided the clinical records to review the selected hospital discharges (any potentially identifiable information was not utilized during the study). Patients with a clinical history of more than one hospitalization in the same year were included in our analysis. Misclassified hospitalizations (patients under 18 years of age and patients for whom the actual diagnosis in the medical records did not correspond to a DM/ACSC) were discarded.

### Data collection

Based on the clinical records, we collected the quantity of each resource used (from ward admission to discharge) in seven cost centers [36]: (1) Length of stay (LoS); (2) Medical and nonmedical consultations; (3) Procedures performed during hospitalization (surgical or nonsurgical interventions); (4) Laboratory studies performed; (5) Medical imaging studies performed; (6) Crystalloid and colloid solutions administered; and (7) Medications administered.

### Unitary costs

LoS includes the number of days each hospitalized patient stayed in each service (e.g., emergency, internal medicine, surgery, and intensive care unit) according to their clinical records. The unit cost corresponds to a bed day, excluding the costs of medical care, medications, and medical supplies. The cost was obtained from the official tabulator cost recovery fee [37–39] and the Guidelines for Service Exchange in the Health Sector [40].

Medical and nonmedical consultations included the number of consultations made by physicians, nurses, nutritionists, and psychologists during hospitalization registered in the clinical records. The consultations included healthcare professional fees. For procedures, all surgical and nonsurgical interventions recorded in nursing sheets, procedure completion sheets, anesthesiology sheets, and surgery sheets were considered, including debridement, mechanical washing, wound dressing, and partial or total amputation of a limb, among others. The unit cost of procedures does not include medicines, laboratory tests, or medical imaging studies. For consultations and procedures, the unitary cost was obtained from the official tabulator cost recovery fee [37–39] and the Guidelines for Service Exchange in the Health Sector [40].

Laboratory and other complementary analyses were grouped based on the type and number of tests performed (e.g., blood chemistry of 2, 3, 4, 5, and 6 elements; liver function tests; blood biometry; coagulation tests; serum electrolytes according to the number of elements analyzed; general urinalysis; blood gas tests; thyroid profile; and cardiac profile). Complementary tests included imaging studies (e.g., tomography, ultrasound, radiography, Doppler) and other methods, such as electrocardiogram and spirometry. The unit cost was obtained from the official tabulator cost recovery fee [37–39], the IMSS purchasing portal [41], the Health Supply Input Control Board [42], and the Guidelines for Service Exchange in the Health Sector [40].

The quantities of the solutions and medications administered were retrieved and counted from the nursing sheets (oral by unit or IV by milliliters). The solutions were categorized into colloids and crystalloids, and in turn, each was subcategorized according to their type and presentation (e.g., globular package, plasma, albumin, saline solution, Hartman solution, glucose). The unitary cost for solutions and medications was obtained from the Guidelines for Acquiring Medicines Associated with the Universal Health Services Catalog and the Protection Fund Against Catastrophic Medical Expenses according to each State’s fees [43], from the IMSS purchasing portal [41], and the ISSSTE’s Health Supplies Input Control Board [42].

Market costs were used as a proxy when no information was available from the institution, which occurred for less than 3% of the resources used during hospitalization.

### Total costs of DM/ACSC hospitalizations

Direct healthcare costs were calculated from the healthcare provider’s perspective using the bottom-up microcosting approach. This approach calculates the total costs of consuming all resources and services performed during the inpatient care process in each hospital [12] and permits the identification of local variations [44].

The total DM/ACSC hospital care cost per hospitalization episode was calculated by adding the costs of all the resources used in each cost center, multiplied by their corresponding unit cost [44]. Overall hospital costs for DM/ACSC were calculated for each institution and DM/ACSC complications using the following ICD-10 codes: coma (E1X.0), ketoacidosis (E1X.1), renal complications (E1X.2), ophthalmic complications (E1X.3), neurological complications (E1X.4), peripheral circulatory complications (E1X.5), other specific complications (E1X.6), multiple complications (E1X.7), complications not specified (E1X.8), and without mention of any complication (E13.9 and E14.9) [16]. We followed the same approach for a standard DM/ACSC hospitalization episode to calculate the total cost of hospital care per episode with a DM/ACSC complication. We added the total costs for all complications to get the overall cost associated with DM/ACSC complications. We then calculated the average cost by dividing the total cost by the number of DM/ACSC hospitalizations for each institution and the complications.

To estimate the total national cost for 2016, we multiplied our average cost per complication and institution by the corresponding number of hospitalizations reported by the Department of Health Statistics [35] for the same year. In cases where our sample lacked data for ICD-10 codes present at the national level, we employed a more conservative cost derived from other institutions for the same code.

Finally, we estimated the percentage of total DM/ACSC costs as a percentage of GDP per capita [45], GDP related to health and social services at the national level according to 2016 data [46], and the percentage of total DM/ACSC costs over total institution hospital care expenses [47].

All costs were converted to constant 2018 United States dollars (USD) based on the OECD conversion factor of 1.04 [48] and the 2016 exchange rate of 18.70 Mexican pesos per USD [49].

## Results

**Fig. 1:**
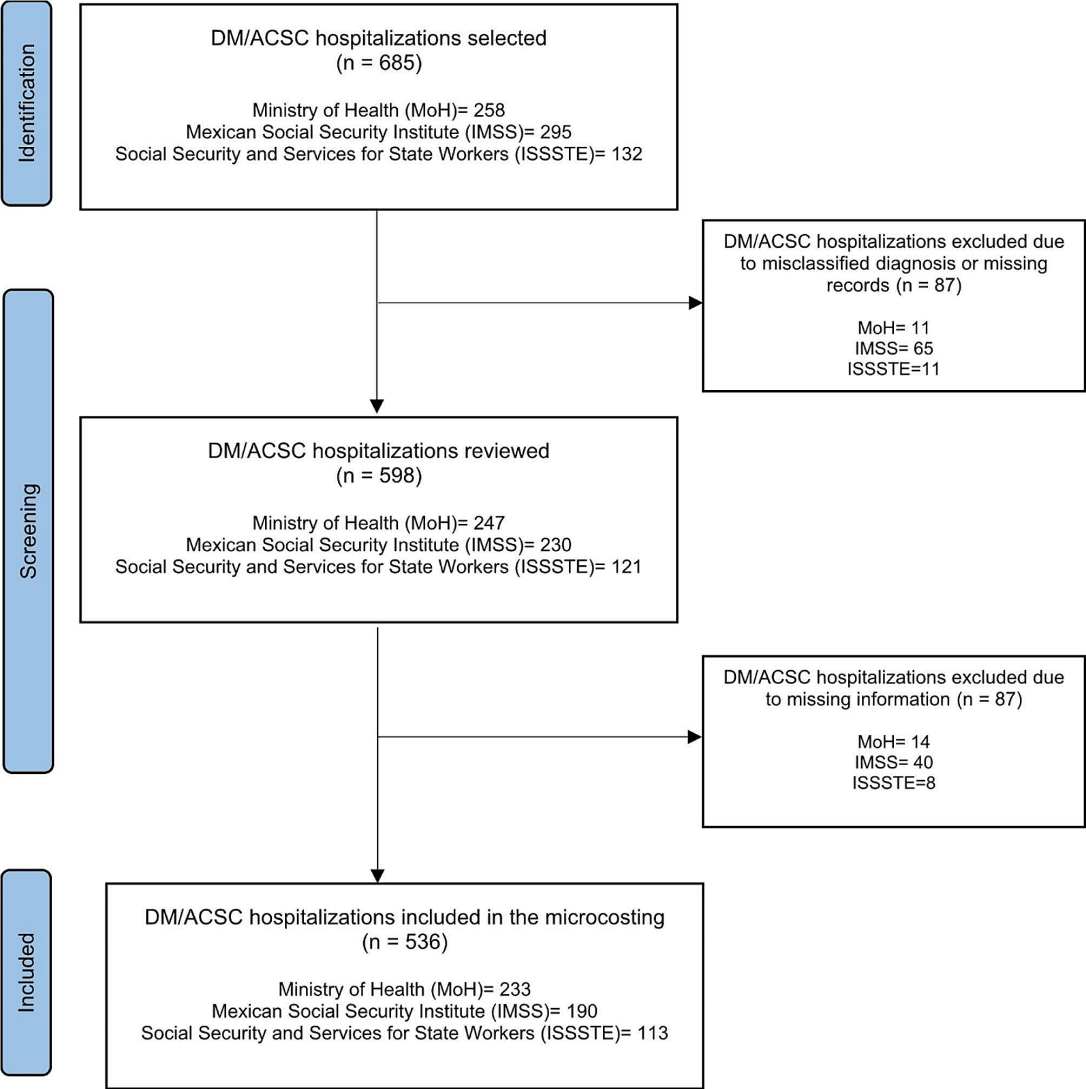
DM/ACSC hospitalizations selected, reviewed, and included in the analysis by institution

In the discharge databases of the selected hospitals, we identified 1,803 DM/ACSC hospitalizations in patients ≥ 18 years old, excluding one hospital in the ISSSTE network that declined to participate in the study. We analyzed a sample of 643 DM/ACSC hospitalizations from 625 clinical records. Of those, 87 hospitalizations were discarded due to problems related to the information system, including incorrect ICD-10 codes that did not correspond to a DM/ACSC diagnosis or patients aged < 18 years. Inconsistencies were more frequent in the IMSS hospitals (Fig. 1). We discarded 22% of the medical records from the IMSS, 12.7% from the ISSSTE, and 4.3% from the MoH facilities.

We reviewed 598 DM/ACSC hospitalizations from 532 medical records, with an average of 1.12 hospitalizations per patient in the same year (the IMSS had an average of 1.26 episodes per patient in the same year). After discarding misclassified cases and those without information about consumed resources, we calculated the direct cost of 536 cases. The sample size per hospital ranged between 43 and 90 (Fig. 1).

### Costs of DM/ACSC hospitalizations by center and institution

Our sample’s average cost of DM/ACSC hospitalization was $1,585. This amount varies across institutions, ranging from $1,427 in the MoH to $1,754 in the ISSSTE. There are differences in the average cost distribution and cost center percentages among the institutions. The average cost per hospitalization for LoS, medicines, and medical imaging studies is higher in the ISSSTE, while consultation and medication costs were lower for the MoH (Table 1).

LoS is the major cost driver for hospitalization across the three institutions. This represents 44.7% of the average cost in the MoH facilities, followed by 38% in the IMSS and 41% in the ISSSTE. Surgical and nonsurgical procedures are the second most expensive cost center, with variations observed between institutions: 25.8% in MoH, 23.8% in IMSS, and 20.7% in ISSSTE (Table 1).

**Table 1:** Average cost (in USD) per DM/ACSC hospitalization by cost center and institution

Cost center	Mexican Public Healthcare Institutions						TOTAL (*n* = 536)	
	MoH (*n* = 233)		IMSS (*n* = 190)		ISSSTE (*n* = 113)			
	Average cost	%	Average cost	%	Average cost	%	Average cost	%
Length of stay	$637	44.7	$637	38.0	$722	41.2	$655	41.3
Procedures	$368	25.8	$400	23.8	$363	20.7	$378	23.9
Consultations	$240	16.8	$352	21.0	$337	19.2	$300	18.9
Laboratory studies	$128	9.0	$138	8.2	$165	9.4	$139	8.8
Medicines	$26	1.8	$109	6.5	$118	6.8	$75	4.7
Medical imaging studies	$23	1.6	$35	2.1	$40	2.3	$31	1.9
Solutions	$5	0.4	$6	0.4	$9	0.5	$7	0.4
Total average cost per hospitalization	$1,427	100	$1,677	100	$1,754	100	$1,585	100

USD = United States dollar; DM/ACSC = diabetes mellitus-related ambulatory care sensitive condition; MoH = Ministry of Health; IMSS = Mexican Social Security Institute; ISSSTE = Institute of Social Security and Services for State Workers

### Cost of DM/ACSC hospitalizations by ICD-10 codes

The average cost per DM/ACSC hospitalization by complication at the three institutions ranged from $463 for a non-specified complication (E1X.6) to $ 2,119 for a peripheral circulatory complication (E1X.4) (Table 2). Peripheral circulatory complications were the most frequent cause of DM/ACSC hospitalization in our sample, and they also had the highest average cost across all institutions. Ketoacidosis (code E1X.1) and renal complications (E1X.2) are the following most frequent and costly complications.

Differences in DM/ACSC hospitalization costs are evident across the institutions, with IMSS facilities incurring higher costs for most complications. However, peripheral circulatory complications are more expensive in the ISSSTE, and neurological complications are more costly in the MoH.

**Table 2:** Average cost (in USD) per DM/ACSC hospitalization by complication and institution

DM/ACSC complication (ICD-10 code)	Mexican Public Healthcare Institutions						TOTAL	
	MoH		IMSS		ISSSTE			
	n	Average cost per DM/ACSC hospitalization	n	Average cost per DM/ACSC hospitalization	n	Average cost per DM/ACSC hospitalization	n	Average cost per DM/ACSC hospitalization
Coma (E1X.0)	10	$618	-	-	-	-	10	$618
Ketoacidosis (E1X.1)	42	$1,248	30	$1,330	29	$1,204	101	$1,261
Renal complication (E1X.2)	5	$1,067	78	$1,235	-	-	83	$1,225
Ophthalmic complication (E1X.3)	15	$405	5	$1,532	1	$104	21	$659
Neurological complication (E1X.4)	3	$890	3	$795	1	$460	7	$788
Peripheral circulatory complication (E1X.5)	123	$1,953	62	$2,279	62	$2,286	247	$2,119
Other specific complication (E1X.6)	-	-	8	$2,972	20	$1,050	28	$1,599
Complication not specified (E1X.8)	23	$462	-	-	-	-	23	$463
Type 2 diabetes without expressed complication (E13.9, E14.9)	12	$741	4	$1,831	-	-	16	$1,014
Multiple complications (E1X.7)	-	-	-	-	-	-	-	-

USD = United States dollar; DM/ACSC = diabetes mellitus-related ambulatory care sensitive condition; ICD-10 = International Classification of Diseases Tenth Revision; MoH = Ministry of Health, IMSS = Mexican Social Security Institute; ISSSTE = Institute of Social Security and Services for State Workers; E1X.0 = E10.0, E11.0, E12.0, E13.0, E14.0; E1X.1 = E10.1, E11.1, E12.1, E13.1, E14.1; E1X.2 = E10.2, E11.2, E12.2, E13.2, E14.2; E1X.3 = E10.3, E11.3, E12.3, E13.3, E14.3; E1X.14 = E10.4, E11.4, E12.4, E13.4, E14.4; E1X.5 = E10.5, E11.5, E12.5, E13.5, E14.5; E1X.6 = E10.6, E11.6, E12.6, E13.6, E14.6; E1X.7 = E10.7, E11.7, E12.7, E13.7, E14.7; E1X.8 = E10.8, E11.8, E12.8, E13.8, E14.8

### Overview of hospitalizations for DM/ACSC in Mexico

In 2016, DM/ACSC caused 115,891 hospitalizations in Mexico’s three major public institutions, with peripheral circulatory and renal complications being the most frequent causes (Table 3). These two complications represent 61% of all DM/ACSC hospitalizations, accounting for almost 70% of the total direct costs. Peripheral circulatory complications alone contribute to half the estimated $178 million total costs caused by DM/ACSC hospitalizations.

In 2016, DM/ACSC hospitalizations represented close to 1% of the GDP related to health and social services at the national level and 2% of total public expenses for general hospital care in Mexico. By institution, the total cost for DM/ACSC hospitalizations was 8.9%, 2.1%, and 1.3% for IMSS, MoH, and ISSSTE, respectively.

**Table 3:** Total direct costs (in USD) of DM/ACSC hospitalizations in Mexico by complication and institution, 2016

DM/ACSC complication (ICD-10 code)	Mexican Public Healthcare Institutions									TOTAL		
	MoH			IMSS			ISSSTE					
	n	n%	Cost	n	n%	Cost	n	n%	Cost	n	n%	Cost
Peripheral circulatory complications (E1X.5)	17,868	34.6	$34,904,069	20,390	38.6	$46,471,959	3600	31.4	$8,229,369	41,858	36.1	$89,605,397
Renal complications (E1X.2)	11,180	21.6	$11,929,065	14,611	27.7	$18,039,100	3096	27.0	$3,303,434*	28,887	24.9	$33,271,599
Complications not specified (E1X.8)	7,021	13.6	$3,394,636	2,544	4.8	$1,230,018*	1470	12.8	$710,741*	11,035	9.5	$5,335,395
Multiple complications (E1X.7)	3,405	6.6	$3,411,518^§^	3,054	5.8	$4,199,523^§^	1260	11.0	$1,385,849^§^	7,719	6.7	$8,996,890^§^
Ketoacidosis (E1X.1)	3,971	7.7	$4,956,649	3,162	6.0	$4,206,321	452	3.9	$544,295	7,585	6.5	$9,707,265
Other specific complications (E1X.6)	3,054	5.9	$3,206,735*	2,536	4.8	$7,537,358	911	8.0	$956,561	6,501	5.6	$11,700,654
Diabetes T2 without expressed complication (E13.9, E14.9)	2,900	5.6	$1,983,2745	2,264	4.3	$2,764,035	228	2.0	$155,926*	5,392	4.7	$4,903,236
Ophthalmic complications (E1X.3)	610	1.2	$247,3145	3,048	5.8	$4,668,727	34	0.3	$3,533	3,692	3.2	$4,919,575
Coma (E1X.0)	1,173	2.3	$725,403	465	0.9	$287,564*	209	1.8	$129,249*	1,847	1.6	$1,142,216
Neurological complications (E1X.4)	477	0.9	$424,545	710	1.3	$533,887	188	1.6	$86,555	1,375	1.2	$1,044,987
TOTAL	51,659	100	$73,450,568	52,784	100	$84,466,713	11,448	100	$19,882,401	115,891	100	$177,798,682

USD = United States dollar; DM/ACSC = diabetes mellitus-related ambulatory care sensitive condition; ICD-10 = International Classification of Diseases Tenth Revision; MoH = Ministry of Health, IMSS = Mexican Social Security Institute; ISSSTE = Institute of Social Security and Services for State Workers; E1X.0 = E10.0, E11.0, E12.0, E13.0, E14.0; E1X.1 = E10.1, E11.1, E12.1, E13.1, E14.1; E1X.2 = E10.2, E11.2, E12.2, E13.2, E14.2; E1X.3 = E10.3, E11.3, E12.3, E13.3, E14.3; E1X.14 = E10.4, E11.4, E12.4, E13.4, E14.4; E1X.5 = E10.5, E11.5, E12.5, E13.5, E14.5; E1X.6 = E10.6, E11.6, E12.6, E13.6, E14.6; E1X.7 = E10.7, E11.7, E12.7, E13.7, E14.7; E1X.8 = E10.8, E11.8, E12.8, E13.8, E14.8.

*Cost estimated using the more conservative cost from other institutions for the condition; § Cost estimated using the weighted average cost of ACSC in each institution

## Discussion

Our study provides evidence of the economic burden of DM/ACSC, both in total and by specific complications, from the health system’s perspective with a bottom-up microcosting approach. Previous studies on general DM care costs [24, 26, 50] have utilized either DRGs [8, 21, 25] or, when detailing inputs, relied on consensus-built ideal healthcare scenarios [24] or service utilization data from population surveys [50]. Additionally, these studies only addressed one [8, 21, 25, 26] or two [50] of the three public institutions included in this study. Their approaches do not allow us to fully understand the cost burden of DM/ACSC for the healthcare system, the variations between institutions, and the key drivers of these costs.

Our methodological approach has several advantages. The detailed microcosting allows a clear breakdown and understanding of costs in each hospital since the data were obtained from each patient for each cost driver (LoS, medication, laboratory and medical imaging studies, procedures, and personnel visits) [12]. On the other hand, the bottom-up approach for obtaining national estimates ensures the capture of local and institutional variations [44].

### Total burden of DM/ACSC hospitalization costs

The economic burden of type 2 DM and its complications has increased rapidly worldwide, including in Mexico [8, 14]. The magnitude differs between and within countries, but the data should be contextualized to understand them better [14]. In the case of Mexico, we found that the cost of DM/ACSC hospitalizations at three institutions contributes to approximately 1% of the GDP related to health and social services and 2% of total public expenses for hospital care. However, the cost varies between institutions. For example, IMSS bears higher costs, both in absolute and relative terms (Table 3). A similar finding was reported in a study that compared costs between public institutions using standardized cases adjusted by type of institution [15]. These differences may be related to structural differences in primary and hospital care access and the process and quality of care [7]. We found that IMSS patients had more hospital episodes in one year than patients at the other institutions (Fig. 1) and that the average cost per hospitalization was lower than that for ISSSTE patients (Table 1). However, the increased use of hospital services related to DM/ACSC makes the economic burden more relevant.

### The noticeable relative importance of some specific complications

Among the three health institutions, a higher cost (close to 70%) and the number of DM/ACSC hospital episodes are linked to two conditions, peripheral circulatory complications, and renal complications, indicating the importance of investing in prevention in the primary healthcare setting, especially for these two DM/ACSC complications. Differences in costing methods and the coding/classification of DM/ACSC in other studies make it difficult to compare results. Despite this, we have found similar results for the IMSS in another study that used ICD-10 codes [25] but was somewhat dissimilar in other studies, not only compared with our results but also among the different studies. According to some studies, the highest cost burden is associated with renal disease [24], cardiovascular disease [50], and retinopathy [51]. These examples demonstrate that different methodologies can be a significant variation driver in calculating the cost of illness [31] and that the cost of illness interpretation needs to consider the particular costing methods used [14]. In this regard, our bottom-up microcosting approach provides a clearer understanding of the characteristics of hospitals’ direct costs by condition and the actual opportunity costs of resources used for DM/ACSC hospital care [14].

### The drivers of inter-institutional variability

Variation between and within countries in ACSC hospital rates and costs is a common finding in ACSC studies [4, 5, 8, 11, 22, 52]. As we have stated above, the analysis of inter-institutional variability may reflect structural differences in the primary and hospital care provided by each institution in terms of processes, resource utilization, and quality of care [7]. Service coverage offered for DM/ACSC hospital care differs by institution. For example, renal complications are covered entirely by the IMSS but not by the MoH. These differences may explain the relatively higher cost burden due to renal complications found in the IMSS, highlighting the context and importance of system characteristics in interpreting hospitalization rates and costs for ACSC [14]. Variations in ACSC hospitalizations cannot be explained only by the characteristics of PHC [2, 6]. A systematic review [4] concluded that PHC quality and hospital care access are drivers of variability in ACSC hospitalization rates. On the other hand, hospital care quality is the main driver of variability in LoS and a significant driver of costs.

Patient characteristics are another potential source of inter-institutional variation. Among those factors, lower socioeconomic status is frequently reported to be associated with higher ACSC hospitalization rates [5, 11, 13, 14, 53, 54], living in rural areas [11], aging [14], and poorer health (including multiple chronic conditions) [11, 55].

Although it is important to note that we do not have precise data about differences in patient characteristics by institution, MoH patients are generally of lower socioeconomic status and, presumably, would have more ACSC hospitalizations and more direct costs. Despite being first in the population covered, the MoH is second after the IMSS in DM/ACSC hospitalizations and costs; this highlights again the importance of system features (the MoH does not provide full coverage for all services related to DM/ACSC complications), probably well above the influence of the characteristics of the patients.

In addition to the inter-institutional variation in the total costs that system characteristics could partially explain, the variability in the average cost and its composition for the same type of patient (Table 1) reveals differences in the care processes at the three institutions. This variability suggests quality of care problems, probable inefficiencies, non-standardization of medical practices, or waste of resources, which would be worth investigating by further research.

### DM/ACSC inpatient care as an opportunity cost in DM care

The cost of LoS was the most crucial input of all main costs for DM/ACSC hospitalizations across the three institutions (Table 1). Research has shown that inpatient ACSC treatment is more expensive than outpatient or emergency department (ED) treatments. ED visits can be 320 to 728% more expensive than PHC visits [12]. Shifting the care from inpatient to outpatient care without compromising quality is a potential strategy to decrease the DM/ACSC cost burden. Studies have revealed significant variations in the decision to admit or not admit patients at the hospital and physician levels. Replacing hospital admissions with ED or outpatient services could be a feasible strategy [12]. However, the potential savings from preventing in-hospital care would be even higher with investments in quality PHC. Costs of ACSC increase over time, and with disease severity, early investment in prevention, a genuine function of PHC, would be worthwhile [14]. PHC quality in general [4] and particular quality features such as continuity of care [3, 6] are significantly related to lower DM/ACSC hospitalization rates. In addition, hospital care quality is pointed out as the primary driver of variability in LoS [4]. Therefore, the cost burden of DM/ACSC inpatient care seems closely related to suboptimal quality care management throughout the health system.

### Limitations

The quality of the information in the hospital discharge databases may have limited the identification of all cases of DM/ACSC in the hospitals included in the study. For example, the under-registrations in the clinical files may have led to underestimating costs, even though the Official Mexican Standard NOM-004-SSA3-2012 indicates the criteria for integrating and quality clinical records. Conversely, in some of the hospitals included in the study, there were no cases of some DM/ACSC complications, probably due to coding problems or the actual absence of patients who could be admitted to other facilities. Furthermore, some unit costs (such as day of hospital stay, consultations, surgical or nonsurgical interventions, laboratory studies, and medical imaging studies) are sourced from official tabulators, which may not accurately capture actual production costs. These issues may lead to a conservative calculation of the total cost of the DM/ACSC. The withdrawal from the study of one ISSSTE hospital could have affected the overall DM/ACSC cost for ISSSTE and the total public system. However, it is unlikely that this could significantly bias the results, given that the estimates for the other ISSSTE hospitals in the study are very similar. ISSSTE covers < 10% of the population, and DM/ACSC hospitalizations are lower than for the other two institutions. Despite these limitations, it should be noted that this is the first attempt to estimate the direct costs generated by DM/ACSC hospitalizations in principal public health institutions in Mexico using data from a primary source, such as clinical records.

## Conclusions

The costs of hospital care for ACSC related to DM represent 1.3–8.9% of the total expense of hospital care by each institution at the national level. Differences in the consumption of resources among hospitals and institutions by the same type of condition are likely the result of the structural characteristics of the institution and perhaps from quality problems in both hospital and primary care, which require further analysis. Our results underline the importance of improving primary care and diabetes management, in general, to reduce preventable hospitalizations. The results of this study suggest that prioritizing enhanced ambulatory care and comprehensive diabetes management holds significant potential for reducing avoidable hospital admissions and associated healthcare costs. Implementing targeted interventions that address identified gaps in care and optimize chronic disease management strategies can lead to substantial cost savings for healthcare systems and improve patient outcomes. Policymakers should prioritize investments in primary care and diabetes management programs to optimize population health and ensure sustainable healthcare systems. Additionally, since peripheral vascular and renal complications represent the highest cost burden, these could be priorities for investing in prevention and quality improvement to increase efficiency.

## Data Availability

The data that support the findings of this study is available in the web portal for hospital discharges of the Mexican Department of Health Statistics [https://datos.gob.mx/busca/dataset/egresos-hospitalarios-de-la-secretaria-de-salud]. The datasets generated and analyzed during the current study are available from the corresponding author upon reasonable request.

## Abbreviations

ACSC: Ambulatory Care Sensitive Conditions
ED: Emergency Department
ICD-10: International Classification of Diseases, Tenth Revision
DM: Diabetes Mellitus
PHC: Primary Health Care
OECD: Organization for Economic Co-operation and Development
MoH: Ministry of Health
IMSS: Mexican Social Security Institute
ISSSTE: Institute of Social Security and Services for State Workers
DRG: Diagnosis-Related Groups
DM/ACSC: Ambulatory Care Sensitive Conditions related to Diabetes Mellitus
INEGI: National Institute of Statistics and Geography
DGIS: Department of Health Statistics
LoS: Length of Stay
GDP: Gross Domestic Product
USD: United States dollar
